# Correction: [^18^F]ROStrace detects oxidative stress in vivo and predicts progression of Alzheimer’s disease pathology in APP/PS1 mice

**DOI:** 10.1186/s13550-023-00963-w

**Published:** 2023-02-27

**Authors:** Chia-Ju Hsieh, Catherine Hou, Yi Zhu, Ji Youn Lee, Neha Kohli, Evan Gallagher, Kuiying Xu, Hsiaoju Lee, Shihong Li, Meagan J. McManus, Robert H. Mach

**Affiliations:** 1 Department of Radiology, Perelman School of Medicine, University of Pennsylvania, Philadelphia, PA 19104 USA; 2 Department of Anesthesiology and Critical Care Medicine, The Children’s Hospital of Philadelphia, Philadelphia, PA 19104 USA; 3 Center for Mitochondrial and Epigenomic Medicine, The Children’s Hospital of Philadelphia, Philadelphia, PA 19104 USA

**Correction to: EJNMMI Research (2022) 12:43** 10.1186/s13550-022-00914-x

Following publication of the original article [1], the authors were notified that 23 of the 91 imaging datasets had not been reconstructed properly. The corrected versions of the 23 mouse images include 11 APP/PS1 (5 mo.: 2 female and 2 male; 16 mo.: 2 female and 5 male) and 12 WT (5 mo. 2 female and 2 male; 16 mo.: 4 female and 4 male). Note that the correction of the imaging datasets make no difference to the conclusions reached in this study. In addition to these corrections, it came to the authors’ attention that acknowledgement of a grant of number NS114656 had been omitted from the Funding declaration; the declaration has since been corrected. The original and the corrected versions of figures and statements in question may be seen below. The original article has been updated with the corrections.

**The original published abstract for the related statement:**

“[^18^F]ROStrace differences emerged mid-life, temporally and spatially correlating with increased Aβ burden (r^2^ = 0.36; *p* = 0.0003), which was also greatest in the female brain (*p* < 0.001).”

**The corrected abstract for the related statement:**

“[^18^F]ROStrace differences emerged mid-life, temporally and spatially correlating with increased Aβ burden (r^2^ = 0.30; *p* = 0.0016), which was also greatest in the female brain (*p* < 0.001).”

**Original published Fig. 1c, d, and e:** 
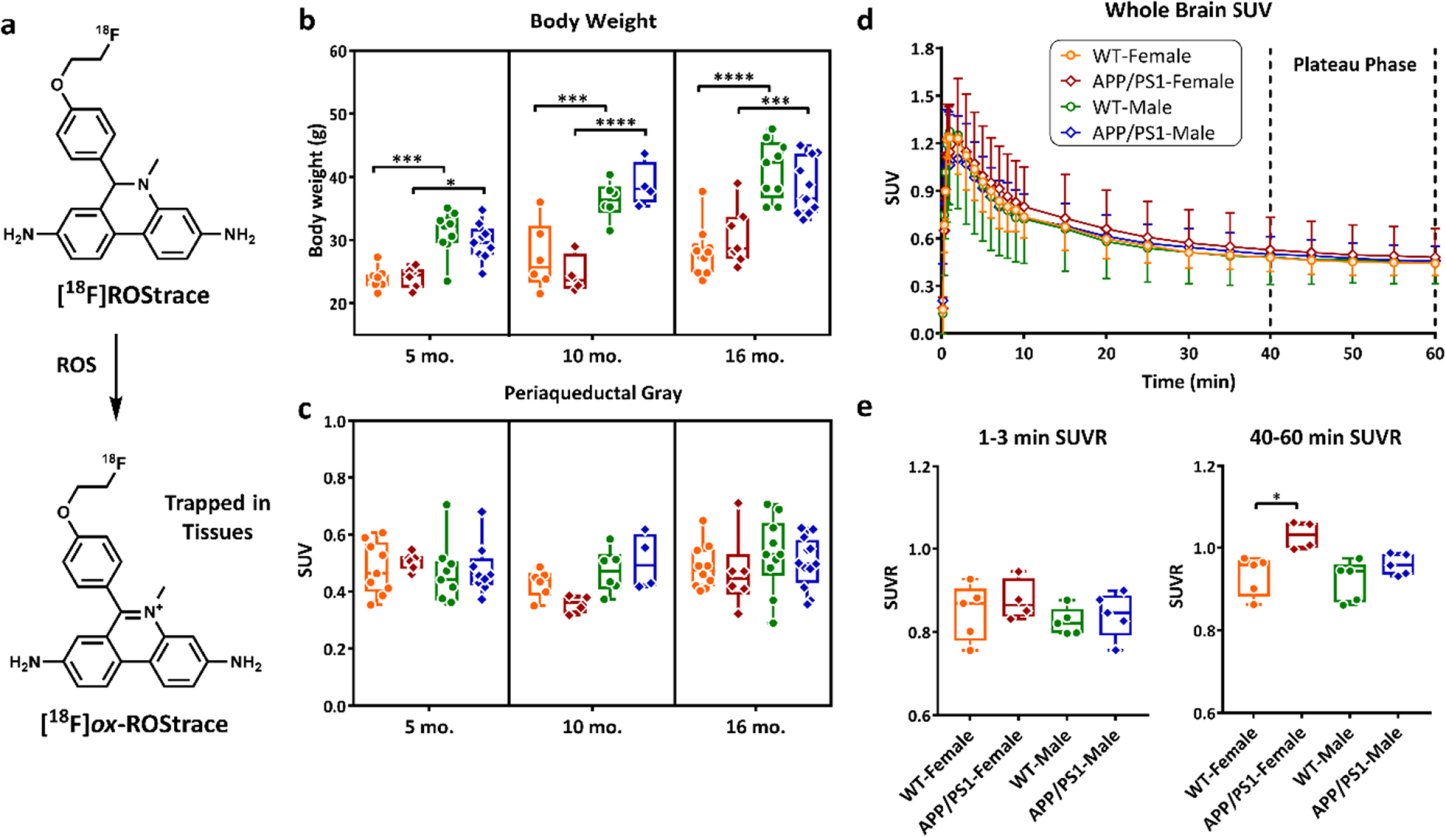


**Corrected Fig. 1c, d, and e:** 
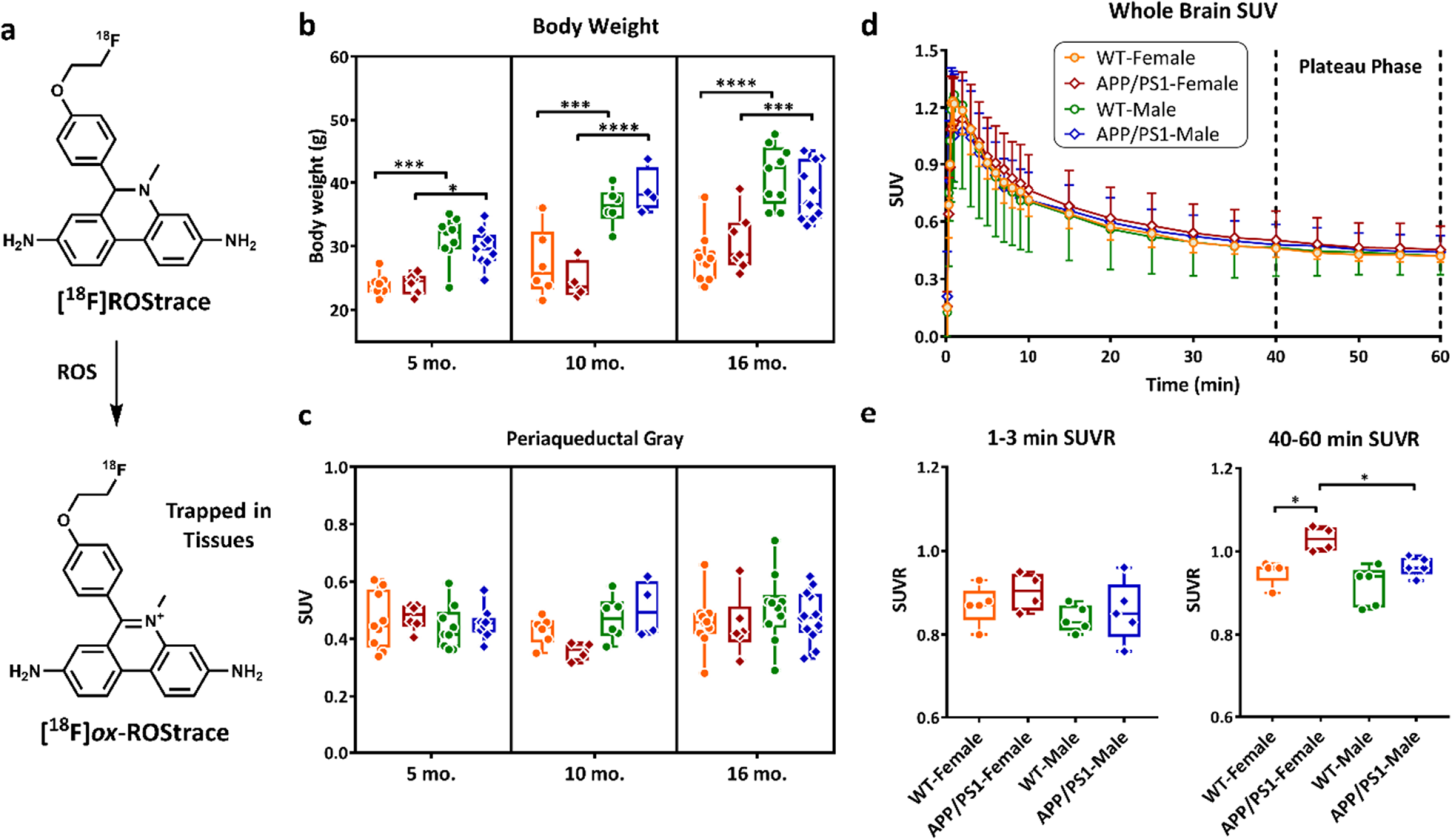


**Original published Fig. 2a and c:** 
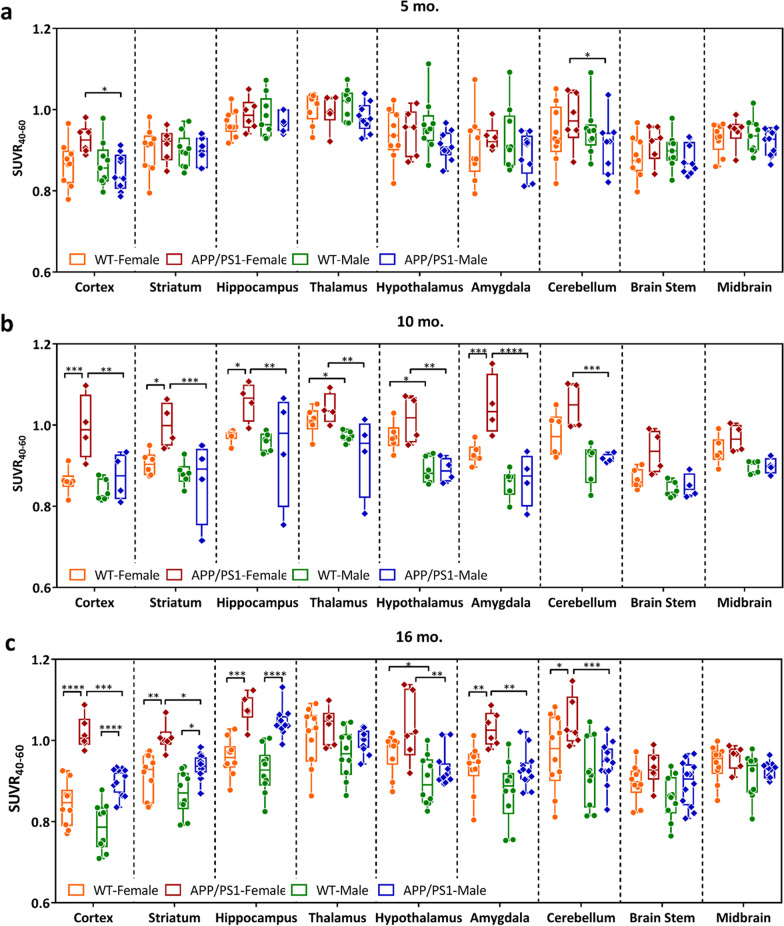


**Corrected Fig. 2a and c:** 
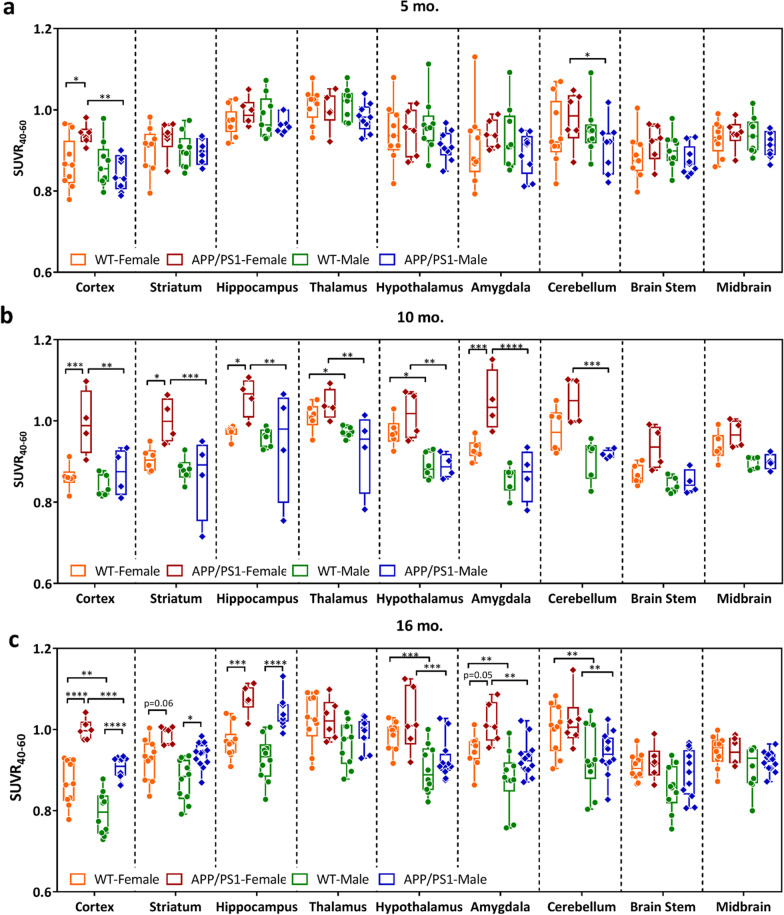


**The original published article for the related Results statement of Fig. 2a and c:**

“The pattern of increased [^18^F]ROStrace retention in female APP/PS1 vs. WT was similar at 10 and 16 mo. of age, but more significant in the cortex (*p* < 0.0001), striatum (*p* = 0.0034), hippocampus (*p* = 0.0001), amygdala (*p* = 0.0031), and cerebellum (*p* = 0.0257) (Fig. 2c, Additional file 1: Table S1). Finally, at 16mo. of age, [^18^F]ROStrace retention increased in APP/PS1 male mice. Higher SUVR40-60 levels were observed in APP/PS1 vs. WT male in cortex (*p* < 0.0001), striatum (*p* = 0.0217), and hippocampus (*p* < 0.0001) (Fig. 2c). Notably, the only region with differential [^18^F]ROStrace retention in female vs. male WT mice was the hypothalamus from mid- (*p* = 0.0156) to advanced (*p* = 0.0165) life.

In order to understand the effect of aging on retention of [^18^F]ROStrace in brain tissue, SUVR40-60 measurements were compared within female and male WT and APP/PS1 mice over time using a two-way ANOVA. The statistical comparisons show a decrease in several regions in male WT mice from 5 to 16 mo., but [^18^F]ROStrace remained stable in most regions of the WT female brain over time (Additional file 1: Table S1). Conversely, [^18^F]ROStrace retention increased over time in mice with APP/PS1 mutations, regardless of sex. From 5 to 16 mo., [^18^F]ROStrace retention increased in the hippocampus (*p* = 0.0055), striatum (*p* = 0.0023), cortex (*p* = 0.0050), hypothalamus (*p* = 0.0204), and amygdala (*p* = 0.0157) of APP/PS1 females, but only the hippocampus (*p* = 0.0018) and cortex (*p* = 0.0296) of APP/PS1 males.”

**The corrected article for the related Results statement of Fig. 2a and c:**

“The pattern of increased [^18^F]ROStrace retention in female APP/PS1 *vs.* WT was similar at 10 and 16 mo. of age, but augmented in the cortex (*p* < 0.0001) and hippocampus (*p* = 0.0003) over time (Fig. 2c, Additional file 1: Table S1). Finally, at 16mo. of age, [^18^F]ROStrace retention increased in APP/PS1 male mice. Higher SUVR_40-60_ levels were observed in APP/PS1 *vs.* WT male in cortex (*p* < 0.0001), striatum (*p* = 0.0183), and hippocampus (*p* < 0.0001) (Fig. 2c). Differential [^18^F]ROStrace retention in female vs. male WT mice began in the hypothalamus at mid-life (*p* = 0.0156), and spread to the cortex (*p* = 0.0055), amygdala (*p* = 0.0006), and cerebellum (*p* = 0.004) in advanced life.

In order to understand the effect of aging on retention of [^18^F]ROStrace in brain tissue, SUVR_40-60_ measurements were compared within female and male WT and APP/PS1 mice over time using a two-way ANOVA. The statistical comparisons show a decrease in several regions in male WT mice from 5 to 16 mo., but [^18^F]ROStrace remained stable in most regions of the WT female brain over time (Additional file 1: Table S1). Conversely, [^18^F]ROStrace retention increased over time in mice with APP/PS1 mutations, regardless of sex. From 5 to 16 mo., [^18^F]ROStrace retention increased in the hippocampus (*p* = 0.0126) and hypothalamus (*p* = 0.0377), of APP/PS1 females, but only the hippocampus (*p* = 0.0002) of APP/PS1 males.”

**Original published Fig. 4a and e:** 
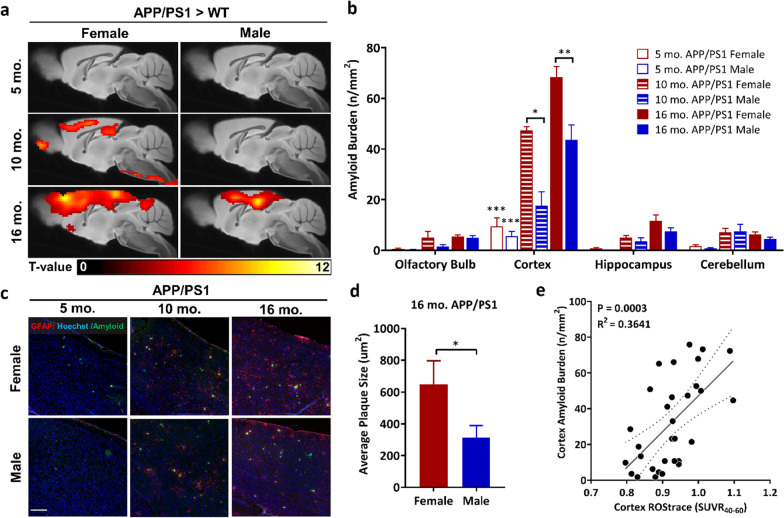


**Corrected Fig. 4a and e:** 
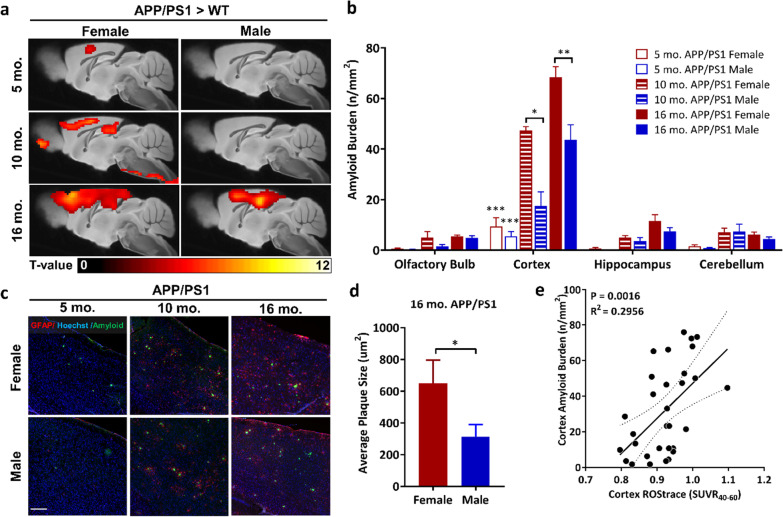


**The original published article for the related Results statement of Fig. 4a and e:**

“To evaluate the significance of this association, linear regression analysis was performed for amyloid burden vs. [^18^F]ROStrace SUVR_40-60_ in the cortex of APP/PS1 mice, which revealed a positive correlation between amyloid burden and [^18^F]ROStrace (*p* = 0.0003, R^2^ = 0.36; Fig. 4e).”

**The corrected article for the related Results statement of Fig. 4a and e:**

“To evaluate the significance of this association, linear regression analysis was performed for amyloid burden vs. [^18^F]ROStrace SUVR_40-60_ in the cortex of APP/PS1 mice, which revealed a positive correlation between amyloid burden and [^18^F]ROStrace (*p* = 0.0016, R^2^ = 0.30; Fig. 4e).”

**The original published article for the related Discussion statement:**

“Interestingly, the only region with significantly different [^18^F]ROStrace retention in WT females and males was the hypothalamus.”

**The corrected article for the related Discussion statement:**

“Interestingly, a significant difference in [^18^F]ROStrace retention in WT females and males was observed in hypothalamus.”

**The original published article for the related Funding statement:**

This research was funded by the National Institutes of Health, National Institute of Aging, grant number AG055142.

**The corrected article for the related Funding statement:**

This research was funded by the National Institutes of Health, National Institute of Aging, grant number AG055142, and the National Institute of Neurological Disorders and Stroke grant number NS114656.

**Original published Table S1:**

**Table:** 

	5 months				10 months				16 months			
	Female		Male		Female		Male		Female		Male	
	Wild type	APP/PS1	Wild type	APP/PS1	Wild type	APP/PS1	Wild type	APP/PS1	Wild type	APP/PS1	Wild type	APP/PS1
Sample size (n)	9	6	9	9	6	4	6	4	10	6	10	12
Cortex	0.87 ± 0.06	0.93 ± 0.04	0.87 ± 0.06	0.85 ± 0.05^**e**^	0.86 ± 0.03	0.99 ± 0.08^**c**^	0.84 ± 0.03	0.87 ± 0.06^f^	0.84 ± 0.05	1.02 ± 0.04^ dl^	0.79 ± 0.06^ l^	0.90 ± 0.03^dgl^
Striatum	0.90 ± 0.06	0.91 ± 0.04	0.90 ± 0.04	0.90 ± 0.04	0.90 ± 0.03	1.00 ± 0.06^ai^	0.88 ± 0.03	0.86 ± 0.10^ g^	0.91 ± 0.05	1.01 ± 0.03^bl^	0.87 ± 0.05	0.94 ± 0.03^aem^
Hippocampus	0.97 ± 0.03	0.99 ± 0.04	0.98 ± 0.05	0.97 ± 0.02	0.97 ± 0.02	1.06 ± 0.05^a^	0.96 ± 0.02	0.95 ± 0.14^f^	0.96 ± 0.04	1.08 ± 0.04^ cl^	0.93 ± 0.06^ k^	1.05 ± 0.03^dln^
Thalamus	1.01 ± 0.04	0.99 ± 0.04	1.01 ± 0.04	0.98 ± 0.04	1.01 ± 0.03	1.04 ± 0.04	0.97 ± 0.01	0.93 ± 0.10^f^	1.01 ± 0.07	1.04 ± 0.04	0.97 ± 0.06	1.00 ± 0.03^ m^
Hypothalamus	0.94 ± 0.06	0.95 ± 0.06	0.96 ± 0.07	0.91 ± 0.04	0.97 ± 0.03	1.01 ± 0.06	0.89 ± 0.03^i^	0.89 ± 0.03^f^	0.97 ± 0.05	1.03 ± 0.08^ k^	0.90 ± 0.06^ek^	0.93 ± 0.04^ g^
Amygdala	0.91 ± 0.08	0.93 ± 0.03	0.93 ± 0.08	0.89 ± 0.05	0.93 ± 0.02	1.05 ± 0.08^cj^	0.86 ± 0.03	0.87 ± 0.07^ h^	0.93 ± 0.06	1.03 ± 0.04^bk^	0.87 ± 0.08	0.93 ± 0.05^f^
Cerebellum	0.94 ± 0.07	0.98 ± 0.07	0.95 ± 0.06	0.90 ± 0.07^**e**^	0.98 ± 0.05	1.05 ± 0.06	0.91 ± 0.05	0.92 ± 0.01^ g^	0.97 ± 0.09	1.05 ± 0.06^a^	0.92 ± 0.08	0.94 ± 0.05^ g^
Brain stem	0.88 ± 0.05	0.92 ± 0.04	0.90 ± 0.04	0.88 ± 0.04	0.87 ± 0.02	0.94 ± 0.05	0.84 ± 0.02i	0.92 ± 0.02	0.90 ± 0.05	0.93 ± 0.04	0.86 ± 0.05	0.89 ± 0.05
Midbrain	0.93 ± 0.04	0.95 ± 0.04	0.94 ± 0.04	0.92 ± 0.03	0.94 ± 0.03	0.97 ± 0.03	0.90 ± 0.01	0.92 ± 0.03	0.94 ± 0.04	0.96 ± 0.03	0.91 ± 0.05	0.93 ± 0.02

**Corrected Table S1:**

**Table:** 

	5 months				10 months				16 months			
	Female		Male		Female		Male		Female		Male	
	Wild type	APP/PS1	Wild type	APP/PS1	Wild type	APP/PS1	Wild type	APP/PS1	Wild type	APP/PS1	Wild type	APP/PS1
Sample size (n)	9	6	9	9	6	4	6	4	10	6	10	12
Cortex	0.87 ± 0.06	0.94 ± 0.03^a^	0.87 ± 0.06	0.85 ± 0.04^f^	0.86 ± 0.03	0.99 ± 0.08^c^	0.84 ± 0.03	0.87 ± 0.06^f^	0.87 ± 0.05	1.00 ± 0.03^d^	0.79 ± 0.05^ fl^	0.90 ± 0.03^dgm^
Striatum	0.90 ± 0.06	0.93 ± 0.04	0.90 ± 0.04	0.90 ± 0.03	0.90 ± 0.03	1.00 ± 0.06^a^	0.88 ± 0.03	0.86 ± 0.10^ g^	0.93 ± 0.05	0.99 ± 0.02	0.87 ± 0.05	0.94 ± 0.03^a^
Hippocampus	0.97 ± 0.04	0.99 ± 0.03	0.98 ± 0.05	0.97 ± 0.03	0.97 ± 0.02	1.06 ± 0.05^a^	0.96 ± 0.02	0.95 ± 0.14^f^	0.97 ± 0.04	1.07 ± 0.03^ck^	0.93 ± 0.05^ k^	1.04 ± 0.04^dln^
Thalamus	1.01 ± 0.04	1.00 ± 0.04	1.01 ± 0.04	0.98 ± 0.04	1.01 ± 0.03	1.04 ± 0.04	0.97 ± 0.01	0.93 ± 0.10^f^	1.02 ± 0.07	1.02 ± 0.05	0.97 ± 0.06	1.00 ± 0.03^ m^
Hypothalamus	0.94 ± 0.07	0.95 ± 0.06	0.96 ± 0.07	0.91 ± 0.04	0.97 ± 0.03	1.01 ± 0.06^i^	0.89 ± 0.03i	0.89 ± 0.03^f^	0.99 ± 0.03	1.02 ± 0.08^ k^	0.90 ± 0.06^gk^	0.93 ± 0.05^ g^
Amygdala	0.91 ± 0.10	0.94 ± 0.03	0.93 ± 0.08	0.89 ± 0.05	0.93 ± 0.02	1.05 ± 0.08^ci^	0.86 ± 0.03	0.87 ± 0.07^ h^	0.95 ± 0.04	1.02 ± 0.05	0.88 ± 0.07^f^	0.93 ± 0.04^f^
Cerebellum	0.94 ± 0.08	0.98 ± 0.07	0.95 ± 0.06	0.90 ± 0.06^e^	0.98 ± 0.05	1.05 ± 0.06	0.91 ± 0.05	0.92 ± 0.01^ g^	1.00 ± 0.06	1.02 ± 0.07	0.93 ± 0.08^f^	0.94 ± 0.05^f^
Brain stem	0.89 ± 0.06	0.92 ± 0.05	0.90 ± 0.04	0.88 ± 0.04	0.87 ± 0.02	0.94 ± 0.05	0.84 ± 0.02i	0.92 ± 0.02	0.91 ± 0.03	0.92 ± 0.04	0.86 ± 0.06	0.89 ± 0.06
Midbrain	0.93 ± 0.04	0.94 ± 0.04	0.94 ± 0.04	0.91 ± 0.03	0.94 ± 0.03	0.97 ± 0.03	0.90 ± 0.01	0.92 ± 0.03	0.95 ± 0.04	0.95 ± 0.03	0.91 ± 0.05	0.92 ± 0.03
