# Supplementary material for: [18F]ROStrace detects oxidative stress in vivo and predicts progression of Alzheimer’s disease pathology in APP/PS1 mice
Source: EJNMMI Res. 2022 Jul 27;12:43. doi: 10.1186/s13550-022-00914-x (PMC9329498; doi:10.1186/s13550-022-00914-x)
Supplement: Supplementary file 1 — Additional file 1: Fig. S1. Schematic illustration of experimental scheme. Figure S2. Comparison of amyloid plaques spatial distribution in age-matched APP/PS1 male and female mouse brains. Figure S3. Oxidative damage detected by 3NT in neurons over time in WT and APP/PS1 cortex. Table S1. Summary of regional SUVR40–60 statistical analysis in age- and gender-matched APP/PS1 and WT mice at the age of 5, 10, and 16 mo. Table S2. Information of antibodies. [file 13550_2022_914_MOESM1_ESM.docx]

**Additional file 1**

**Supplemental Figures**

**
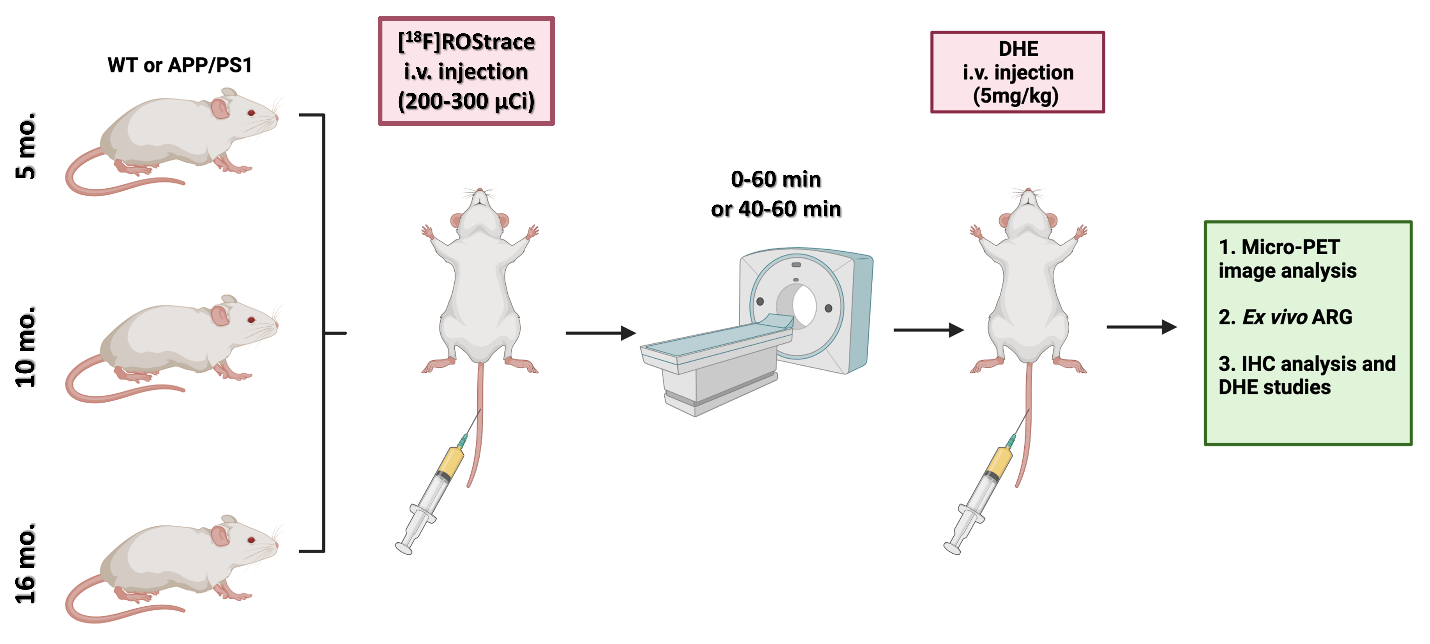
**

**Fig S1.** Schematic illustration of experimental scheme**.** DHE: dihydroethidium; ARG: autoradiography.


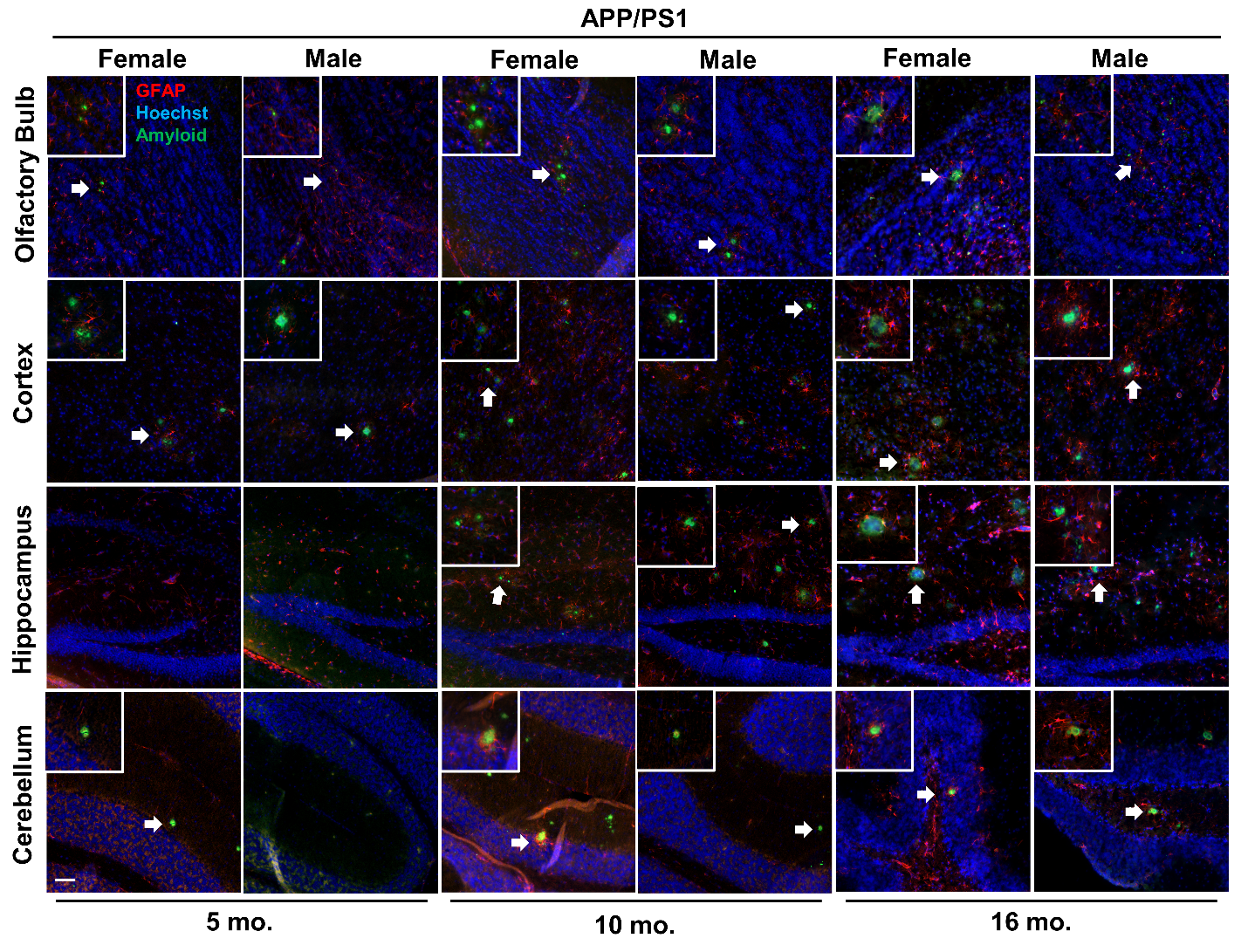


**Fig. S2** Comparison of amyloid plaques spatial distribution in age-matched APP/PS1 male and female mouse brains. Micrographs of 4 key regions from age-matched WT and APP/PS1 brain (olfactory bulbs (top row), cortex (second row), hippocampus (third row) and cerebellum (bottom row)) labeling A$\beta$ (green), astrocytes (red), and nuclei (blue). Inlets are magnified (20x) regions of that indicated by white arrows. Scale bar = 100 μm.

**
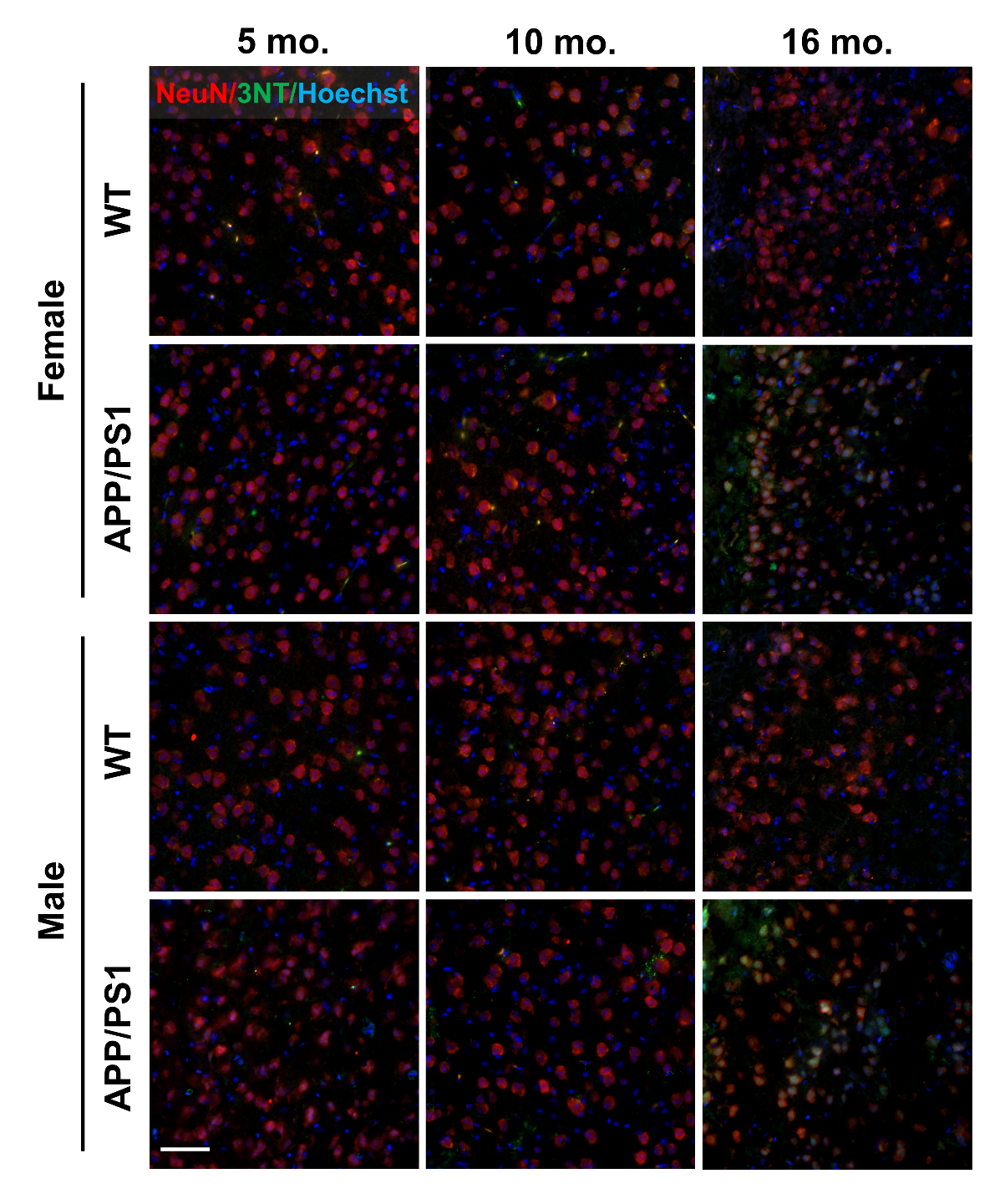
**

**Fig. S3** Oxidative damage detected by 3NT in neurons over time in WT and APP/PS1 cortex. Images of cortex region from age and gender-matched WT and APP/PS1 mouse brain tissues stained with anti-3NT (green) NeuN (red) and Hoechst (blue). Scale bar = 50 μm.

**Table S1.** Summary of regional SUVR_40-60_ statistical analysis in age and gender- matched APP/PS1 and WT mice at the age of 5, 10, and 16 mo.

|  | **5 months** | | | |  | **10 months** | | | |  | **16 months** | | | | |
| --- | --- | --- | --- | --- | --- | --- | --- | --- | --- | --- | --- | --- | --- | --- | --- |
|  | Female | | Male | |  | Female | | Male | |  | Female | | Male | | |
|  | Wild Type | APP/PS1 | Wild Type | APP/PS1 |  | Wild Type | APP/PS1 | Wild Type | APP/PS1 |  | Wild Type | APP/PS1 | Wild Type | APP/PS1 |  |
| Sample Size (n) | 9 | 6 | 9 | 9 |  | 6 | 4 | 6 | 4 |  | 10 | 6 | 10 | 12 |  |
| Cortex | 0.87±0.06 | 0.93±0.04 | 0.87±0.06 | 0.85±0.05**^e^** |  | 0.86±0.03 | 0.99±0.08**^c^** | 0.84±0.03 | 0.87±0.06^f^ |  | 0.84±0.05 | 1.02±0.04^dl^ | 0.79±0.06^l^ | 0.90±0.03^dgl^ |  |
| Striatum | 0.90±0.06 | 0.91±0.04 | 0.90±0.04 | 0.90±0.04 |  | 0.90±0.03 | 1.00±0.06^ai^ | 0.88±0.03 | 0.86±0.10^g^ |  | 0.91±0.05 | 1.01±0.03^bl^ | 0.87±0.05 | 0.94±0.03^aem^ |  |
| Hippocampus | 0.97±0.03 | 0.99±0.04 | 0.98±0.05 | 0.97±0.02 |  | 0.97±0.02 | 1.06±0.05^a^ | 0.96±0.02 | 0.95±0.14^f^ |  | 0.96±0.04 | 1.08±0.04^cl^ | 0.93±0.06^k^ | 1.05±0.03^dln^ |  |
| Thalamus | 1.01±0.04 | 0.99±0.04 | 1.01±0.04 | 0.98±0.04 |  | 1.01±0.03 | 1.04±0.04 | 0.97±0.01 | 0.93±0.10^f^ |  | 1.01±0.07 | 1.04±0.04 | 0.97±0.06 | 1.00±0.03^m^ |  |
| Hypothalamus | 0.94±0.06 | 0.95±0.06 | 0.96±0.07 | 0.91±0.04 |  | 0.97±0.03 | 1.01±0.06 | 0.89±0.03^i^ | 0.89±0.03^f^ |  | 0.97±0.05 | 1.03±0.08^k^ | 0.90±0.06^ek^ | 0.93±0.04^g^ |  |
| Amygdala | 0.91±0.08 | 0.93±0.03 | 0.93±0.08 | 0.89±0.05 |  | 0.93±0.02 | 1.05±0.08^cj^ | 0.86±0.03 | 0.87±0.07^h^ |  | 0.93±0.06 | 1.03±0.04^bk^ | 0.87±0.08 | 0.93±0.05^f^ |  |
| Cerebellum | 0.94±0.07 | 0.98±0.07 | 0.95±0.06 | 0.90±0.07**^e^** |  | 0.98±0.05 | 1.05±0.06 | 0.91±0.05 | 0.92±0.01^g^ |  | 0.97±0.09 | 1.05±0.06^a^ | 0.92±0.08 | 0.94±0.05^g^ |  |
| Brain stem | 0.88±0.05 | 0.92±0.04 | 0.90±0.04 | 0.88±0.04 |  | 0.87±0.02 | 0.94±0.05 | 0.84±0.02i | 0.92±0.02 |  | 0.90±0.05 | 0.93±0.04 | 0.86±0.05 | 0.89±0.05 |  |
| Midbrain | 0.93±0.04 | 0.95±0.04 | 0.94±0.04 | 0.92±0.03 |  | 0.94±0.03 | 0.97±0.03 | 0.90±0.01 | 0.92±0.03 |  | 0.94±0.04 | 0.96±0.03 | 0.91±0.05 | 0.93±0.02 |  |

- *Significance of Wild type and APP/PS1 comparison in male or female by two-way ANOVA: a: p<0.05; b: p<0.01; c: p<0.005; d: p< 0.0001*
- *Significance of male and female comparison in wild type or APP/PS1 by two-way ANOVA: e: p<0.05; f: p<0.01; g: p<0.005; h: p<0.0001*
- *Significance of 5 months and 10 months comparison in wild type male, wild type female, APP/PS1 male or APP/PS1 female by two-way ANOVA: i: p<0.05; j: p<0.01*
- *Significance of 5 months and 16 months comparison in wild type male, wild type female, APP/PS1 male or APP/PS1 female by two-way ANOVA: k: p<0.05; l: p<0.01*
- *Significance of 10 months and 16 months comparison in wild type male, wild type female, APP/PS1 male or APP/PS1 female by two-way ANOVA: m: p<0.05; n: p<0.01*

**Table S2.** Information of antibodies

| **Name** | **Species** | **Cat number** | **Supplier** | **Application** |
| --- | --- | --- | --- | --- |
| Anti-Glial Fibrillary Acidic Protein (GFAP) | Rabbit | AB5804 | Millipore | IF: 1:200 in 1% BSA in PBS |
| Anti-Nitrotyrosine | Rabbit | AB5411 | Millipore | IF: 1:100 in 1% BSA in PBS |
| Anti-beta Amyloid 1-42 | Rabbit | AB180956 | Abcam | IF: 1:200 in 1% BSA in PBS |
| IBA-1 | Goat | AB5076 | Abcam | IF: 1:200 in 1% BSA in PBS |
| IBA-1 | Rabbit | 019-19741 | Wako | IF: 1:200 in 1% BSA in PBS |
| NeuN | Rabbit | ab104224 | Abcam | IF: 1:200 in 1% BSA in PBS |
| Alexa Fluor 488 | Goat | A11008 | Thermo Fisher Scientific | IF: 1:200 or 1:300 in 1% BSA in PBS |
| Alexa Fluor 568 | Goat | A11011 | Thermo Fisher Scientific | IF: 1:300 in 1% BSA in PBS |
| Alexa Fluor 647 | Goat | A21235 | Thermo Fisher Scientific | IF: 1:300 in 1% BSA in PBS |
| Alexa Fluor 594 | Donkey | A11058 | Thermo Fisher Scientific | IF: 1:300 in 1% BSA in PBS |
